# Multivalent Allylammonium-Based Cross-Linkers for the Synthesis of Homogeneous, Highly Swelling Diallyldimethylammonium Chloride Hydrogels

**DOI:** 10.3390/gels8020100

**Published:** 2022-02-08

**Authors:** Tim B. Mrohs, Oliver Weichold

**Affiliations:** Institute of Building Materials Research, RWTH Aachen University, Schinkelstraße 3, 52062 Aachen, Germany; mrohs@ibac.rwth-aachen.de

**Keywords:** hydrogel, copolymer, reactivity ratios, DADMAC, gelation, synthesis

## Abstract

*N*,*N’*-methylenebisacrylamide (BIS) is a very popular cross-linker for the radical polymerisation in water. It is highly reactive but prone to alkaline hydrolysis and suffers from a low solubility. This study shows that with slow polymerising systems such as *N*,*N*-diallyldimethylammonium chloride, only inhomogeneous networks are formed. As a consequence, gels with very low cross-linking densities, i.e., high swelling capacities, disintegrate during the swelling test and firm, coherent gels are not accessible due to the solubility limit. A promising alternative are multivalent tetraallyl-based compounds, of which tetraallylammonium bromide (TAAB), *N*,*N*,*N*’,*N*’-tetraallylpiperazinium dibromide (TAPB) and *N*,*N*,*N*’,*N*’-tetraallyltrimethylene dipiperidine dibromide (TAMPB) are the subject of this study. With these, the cross-linking polymerisation appears to be statistical, as gels formed at low monomer conversion have essentially the same swelling properties as those formed at high conversions. This is not observed with BIS. However, gelation with the tetraallyl cross-linkers is much slower than with BIS and follows the order TAPB < TAMPB < TAAB, but the differences become significantly smaller with increasing content. At low contents, all three allow the preparation of gels with high swelling capacities of up to 360 g/g.

## 1. Introduction

Hydrogels are crosslinked polymer networks which can absorb water from the environment, while maintaining their three-dimensional structure [1]. Hydrogels are divided into two classes, namely ionic and non-ionic hydrogels [2,3]. These two classes differ not only in their molecular structure, but also in their swelling behaviour and the nature of the intermolecular interactions [4]. The swelling characteristics of non-ionic hydrogels are highly dependent on the cross-linker concentration, but are only minimally affected by the salt concentration and the pH value of the surrounding medium [5]. These hydrogels are, therefore, often used in biomedical applications [6] or protein analysis [7]. For ionic hydrogels, the driving forces for swelling in water are the repulsive interactions between the individual charges of the polyelectrolyte chains. Hence, ionic hydrogels usually show a significantly higher swelling capacity compared to non-ionic hydrogels [8]. The synthesis of such hydrogels is usually very simple and inexpensive, so that this class of hydrogels is increasingly used in industrial applications. Some examples are poly (sodium acrylate) as superabsorbers for diapers [9], ion exchange resins for the extraction of toxins such as arsenate [10], or for the reduction of the autogeneous shrinking during the hardening of concrete [11]. A highly alkaline hydrogel for the rehabilitation of reinforced concrete was first introduced in 2018 [12]. This particular hydrogel was synthesised via free radical polymerisation of diallyldimethylammonium hydroxide (DADMAOH), a comonomer and *N*,*N*’-methylenebisacrylamide (BIS) as cross-linker. The gel itself forms a stationary cationic polyelectrolyte backbone with mobile hydroxide counterions. This mobility allows the hydroxide ions in the gel to be exchanged with the carbonate in old concrete, which is a common cause for corrosion of the steel reinforcement. Overall, the cementitious matrix is “realkalised”. This protects the reinforcement from further corrosion. A similar hydrogel was recently used as coupling material for electrochemical chloride extraction [13], an excellent method to counteract chloride-induced steel corrosion. However, the use of BIS as cross-linker in these allyl-based systems can be precarious. As an amide, it is potentially liable to alkaline hydrolysis at prolonged application times, which then produces toxic compounds such as formaldehyde [14]. As an acrylate derivative, it polymerises faster than the notoriously slow allyl compounds. In addition, BIS has a comparatively low solubility of only 20 g/L in water at 20 °C, which makes it less versatile when aiming for firmer gels. On the other hand, the diallylammonium subunit offers a number of synthetic possibilities to prepare cationic and, hence, water-soluble cross-linkers. For example, tetraallylammonium bromide was successfully polymerised with vinylpyrrolidone to give a hydrolytically stable hydrogel which was used to remove dyes from aqueous solutions [15]. *N*,*N*,*N*’,*N*’-tetraallyl piperazinium dichloride, which is resistant to acidic hydrolysis, was used to cross-link acrylamide and acrylic acid [16,17]. Furthermore, a copolymer of *N*,*N*,*N*’,*N*’-tetraallyl trimethylene dipiperidine dichloride with diallyldimethylammonium chloride (DADMAC) was used as a catalytically active ion exchange resin to convert phenols into aromatic ethers [18].

Although some allyl-based cross-linkers are known, the gel properties such as the rheological or swelling properties are largely unexplored, and an evaluation of the cross-linker performance is missing. For a deeper understanding of the important class of cationic hydrogels, these are important parameters. The goal of this study is, therefore, to assess the performance of three quaternary, tetraallylammonium-based cross-linkers, namely tetraallylammonium bromide (TAAB), *N*,*N*,*N*’,*N*’-tetraallylpiperazinium dibromide (TAPB) and *N*,*N*,*N*’,*N*’-tetraallyltrimethylene dipiperidine dibromide (TAMPB) and gels made with the neutral monomer diallyldimethylammonium chloride (DADMAC). These gels are then compared with those containing *N*,*N*’-methylenebisacrylamide (BIS).

## 2. Results and Discussion

**Figure 1:**
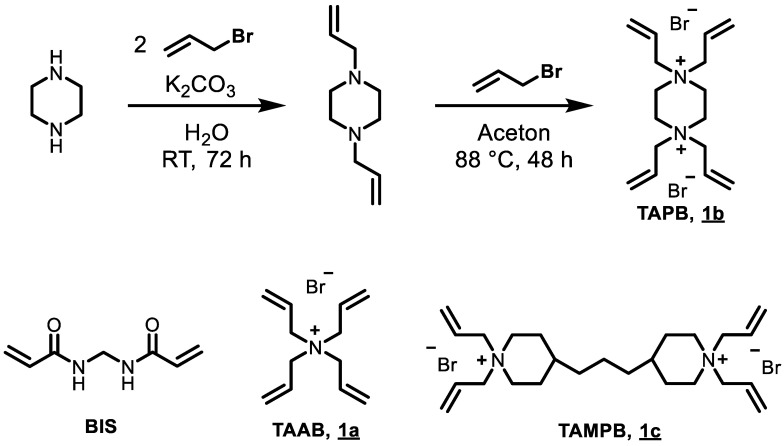
Top: Outline of the two-step synthesis of 1b, c using 1b as example; bottom: structures of the other three cross-linkers used in this study.

The tetraallyl-based cross-linkers tetraallyl ammonium bromide (TAAB, 1a), *N*,*N*,*N*’,*N*’-tetraallyl piperazinium dibromide (TAPB, 1b) and *N*,*N*,*N*’,*N*’-tetraallyl trimethylene dipiperidine dibromide (TAMPB, 1c) were synthesised by allylation of the appropriate amines. However, exhaustive allylation of the secondary amines in the presence of KOH or K_2_CO_3_ was not found to be a viable method. The products from these reactions contain double-digit percentages of KBr, which can only be removed at great loss of product. It is far better to start from the tertiary amines. In the case of TAAB, this is commercially available, and for TAMP and TAPB they can be made from the commercial secondary amines using only a slight excess of allyl bromide. The tertiary amines separate readily from the aqueous reaction mixture and are obtained in sufficient purity to be able to be converted into the quaternary ammonium salts in a subsequent step. The procedure is exemplified for TAPB (1b) in Figure 1.

Following the previously published procedure for cross-linking the polymerisation of DADMAOH with BIS [12], the combination potassium peroxodisulphate (K_2_S_2_O_8_, KPS) and sodium disulphite (Na_2_S_2_O_5_) was chosen as a redox initiation system [19,20], since it was found to be very reliable in this system. In the initial experiments using 2 mol% cross-linker based on DADMAC, gels could be obtained with all four cross-linkers shown in Figure 1. To get more insight into the copolymerisation, the reactivity ratios were calculated using the method of Alfrey and Price [21], Unlike the reactivity ratios, which are only defined for a pair of monomers, Alfrey and Price introduced semiempirical parameters for the reactivity (*Q*) and polarity (*e*) of individual monomers. The reactivity ratios *r*_1,2_ of an unknown pair of monomers can then be calculated from *Q*_1,2_ and *e*_1,2_ according to e. g.,

$$r_{1}=\frac{Q_{1}}{Q_{2}}e^{-e_{1}\left(e_{1}-e_{2}\right)}$$

**Figure 2:**
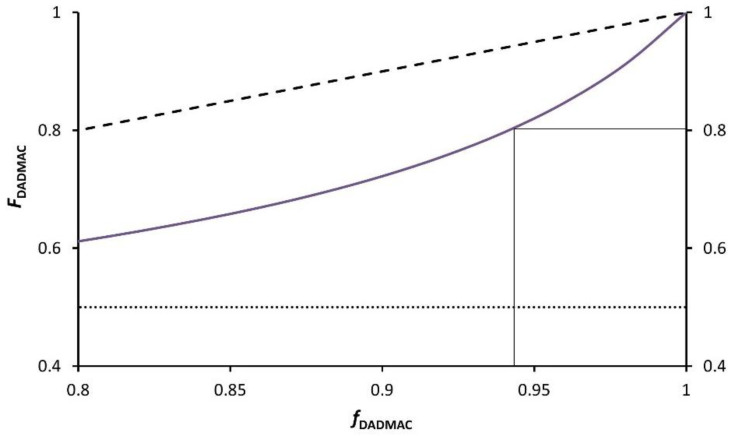
Extract of the calculated copolymerisation diagram of DADMAC with acrylamide, which is used as a substitute for BIS (the full diagram in shown in Figure S16 in the Supporting Information). The solid line indicates the calculated mole fraction of DADMAC in the polymer *F*_DADMAC_ as function of the mole fraction in solution *f*_DADMAC_. The dashed line shows the polymer composition for the statistical copolymerisation and the dotted line the alternating type. The latter are for reference only. The marks indicate the example containing 3 mol% BIS mentioned in the text.

For DADMAC, the parameters *Q*_1_ = 0.32, *e*_1_ = −0.22 are known in the literature [12]. For BIS, these parameters are not known and, thus, acrylamide with *Q*_2_ = 1.18 and *e*_2_ = 1.30 was used as a substitute, assuming a similar polarity *Q* and reactivity *e* [22]. From these, the reactivity ratios *r*_1_  =  0.194 and *r*_2_  =  0.511 can be calculated. That is, both monomers, when added to the chain end, react favorably with the other monomer although this is more pronounced for DADMAC than for BIS. The product *r*_1_   *r*_2_ = 0.099 indicates a certain tendency towards alternating copolymerisation. In the copolymerisation diagram (Figure 2) this can be seen as deviation from the statistical (dashed line) towards the alternating (dotted line) copolymerisation. In total, BIS is incorporated faster into the polymer than DADMAC.

In an assumed monomer solution containing 3 mol% BIS, the effective mole fraction of acrylamide groups in solution is *f*_BIS_ = $\frac{2\cdot \left[BIS\right]}{\left[BIS\right]+\left[DADMAC\right]}$ = 0.057, and as a consequence *f*_DADMAC_ = 1 − *f*_BIS_ = 0.943. As can be seen from Figure 2, chains formed at low conversion (i.e., with little change in the solution composition) contain a mole fraction of DADMAC in the polymer *F*_DADMAC_ = 0.803, and therefore approx. 20 mol% BIS (cf. marking lines in Figure 2). This quickly depletes the reaction mixture of BIS so that the network formed at higher conversion exhibits a significantly lower cross-linking density. In contrast, assuming that the allyl-based cross-linkers 1a–c have reactivity ratios similar to DADMAC, these would be incorporated statistically, i. e., *f* = *F* at any composition (dashed line in Figure 2), thus forming a homogeneous network.

**Figure 3:**
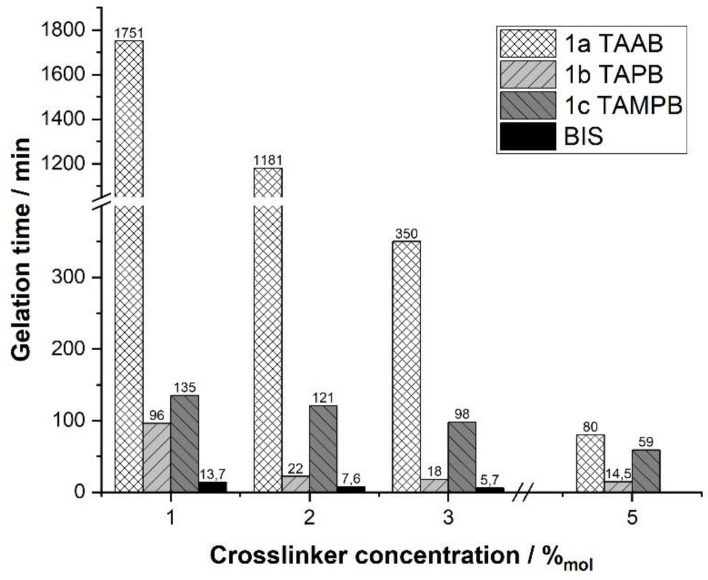
Gelation time of the cross-linking copolymerisation of DADMAC at 20 °C as a function of the cross-linker content. Reproducibility of the gel points is approx. ±10 %.

When qualitatively assessing the gels, the ones containing TAAB (1a) were clearly softer than those containing 1b, c or BIS. To get more insight into the gelation process, the reactions were followed using dynamic rheological measurements that provided the storage and loss modulus as a function of time. In addition, the cross-linker content was varied in the range of 1 to 5 mol%. Mixtures containing 4 mol% of BIS could not be analysed due to the fast gelation, and also turn out to be extremely crumbly; higher BIS contents are inaccessible due to the solubility limit. Gels with high contents of the tetraallyl cross-linkers range from pastelike (TAAB) to firm (TAPB), but coherent. The gelation time, defined as the time of intersection of the storage and loss-modulus curves, decreases for all cross-linkers with increasing concentration, because more cross-linking points are formed in a shorter time at the same polymerisation rate (Figure 3). This way, the elastic properties of the systems develop faster. For all cross-linker contents, the gelation times follow the order BIS < TAPB (1b) < TAMPB (1c) < TAAB (1a), although the differences decrease with increasing content.

**Figure 4:**
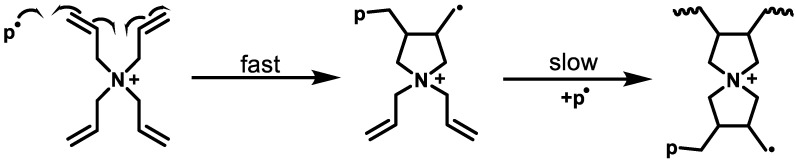
Ring closing reaction of the polymerisation of TAA^+^.

It is remarkable that the gelation times of samples containing TAAB (1a) are considerably longer than those containing TAPB or TAMPB. Similar to DADMAC, for the cross-linkers 1a–c the addition of a radical to one of the allylic double bonds leads to the formation of a 5-membered ring by a 5-exo-dig attack. However, in the case of TAAB (1a) the addition of a second radical, which causes cross-linking of the polymer chains, forms a strained spiro[4,4] structure (Figure 4). It is therefore assumed that this is the rate-determining step for gelation with 1a and it appears to be rather slow. In contrast, cross-linking using TAMPB and TAPB gives rise to spiro[4,5] compounds. This reduces the strain at the spiro centre, because the six-membered ring allows a larger bond angle and the gelation is, therefore, assumed to be faster. This assumption is corroborated by observations made by Blicke and Hotelling during the synthesis of spiro[4,4] and spiro[4,5] ammonium compounds. Under similar conditions, the former is obtained in a 45% yield, while the latter is obtained in a 98% yield [23]. Generally, all three tetraallyl cross-linkers are slower than BIS. Besides the less reactive allylic double bond, the formation of spiro compounds might also contribute to this observation.

**Figure 5:**
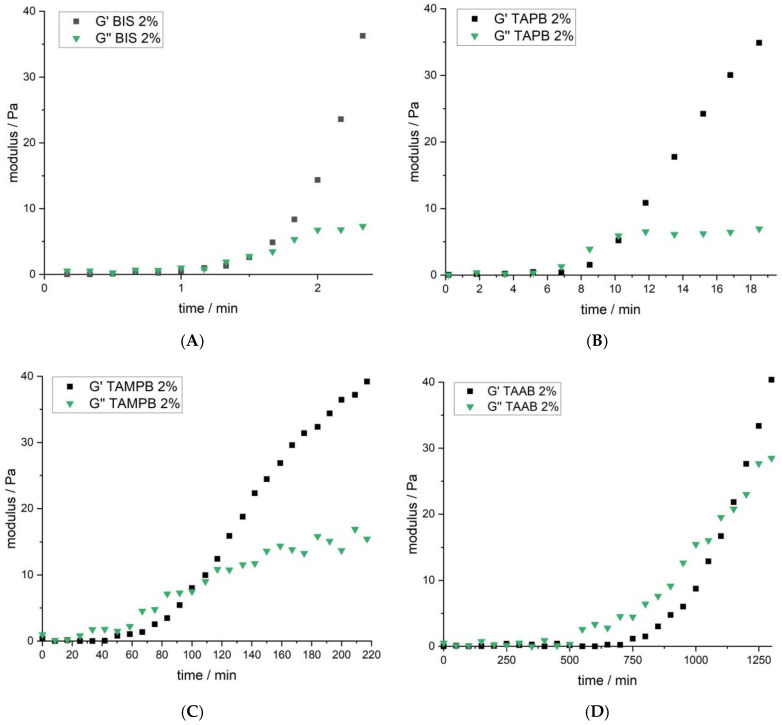
Storage G’ and loss modulus G’’ as a function of time for mixtures containing DADMAC and 2 mol% of the four cross-linkers (**A**) BIS, (**B**) TAPB, (**C**) TAMPB and (**D**) TAAB shown in Figure 1 at 20 °C, recorded at an amplitude of 1 Hz and 1% deformation. Note the different scale of the x-axes.

Further evidence for the slow cross-linking reaction when using TAAB 1a is gained by comparing the evolution of the storage *G*’ and loss moduli *G*’’ of all four polymerisations (Figure 5). The storage modulus *G*’ indicates the elastic properties of the mixture, which is mainly influenced by the cross-linking, while the loss modulus *G*’’ represents viscous properties associated with the irreversible displacement of the free chains.

As can be seen from the copolymerisation diagram, gels made with BIS incorporate a disproportionate amount of cross-linker early in the polymerisation, which leads to a rapid build-up of *G*’ (high cross-linking density), while *G*” increases only marginally before the gel point (Figure 5A). After that, *G*” remains almost constant, while G’ continues to increase rapidly. Similar trends can be observed for TAPB (1b, Figure 5B) and TAMPB (1c, Figure 5C), albeit at extended times, and in the case of 1c at notably higher moduli. This is due to the larger and more flexible structure of TAMPB (1c), which introduces more degrees of freedom and a higher flexibility to the network compared to BIS and 1b. Of the three cationic cross-linkers, TAAB (1a) is expected to have the most rigid structure. However, at the gel point, *G*” has already climbed to more than 20 N m^−2^ (Figure 5D), almost twice the value for 1c and almost 4 times the value of 1b. This is indicative of a much larger number of free polymer chains or longer chain segments connecting the cross-links in mixtures containing 1a as opposed to 1b, c or BIS. In addition, *G*” is not constant after the gel point but still increases, which suggests that even more free chains are formed. Compared to the other three cross-linkers, *G*’ also increases more slowly. Both features account for the previously mentioned softer texture of gels containing 1a and support the mechanism proposed in Figure 4.

^1^H- and ^13^C-NMR spectra of copolymer gels containing TAAB (1a) swollen in D_2_O were not decisive, as these vinyl signals partially overlap with the broad signals of the DADMA^+^ backbone, and the cross-linker concentrations are generally rather small (Figures S17 and S18 in the Supporting Information). Double-bond signals found in the ^13^C-NMR spectra could be addressed to the unreacted DADMAC monomer. However, the TAAB homopolymer clearly showed double-bond signals in the ^1^H-NMR spectrum (Figure S19 in the Supporting Information), which are not identical with the TAAB monomer. The only other explanation is unilaterally incorporated TAAB units. It can be deduced from the integrals that every sixth to seventh unit was only half-incorporated. For the homopolymers of TAPB (1b) and TAMPB (1c), the double-bond signals are significantly lower. This indicates a more homogeneous reactivity of both crosslinker sides. Clearly, this cannot be directly transferred to the cross-linking polymerisation of DADMAC with TAAB, but further supports the above indications for a slow second cyclisation of TAAB, as outlined in Figure 4.

**Figure 6:**
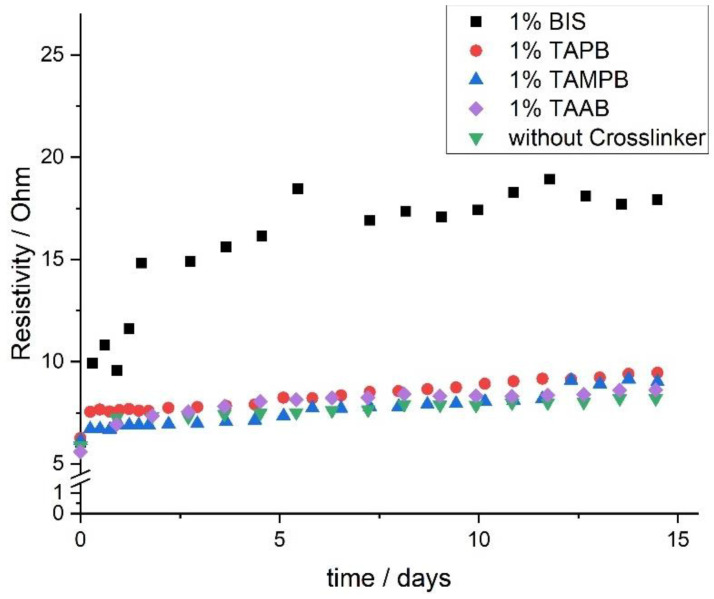
Evolution of the solution resistance *Z*_real_ of pure poly(DADMAC) (▼) and gels cross-linked with 1 mol% BIS, TAAB (1a), TAPB (1b), or TAMPB (1c) over the course of 14 days.

Another important point in time is the end of the reaction. The polymerisation for up to 48 h on the rheometer showed no significant slowdown of the reaction rate based on the increase of *G*’ and the complex viscosity. Monitoring over longer periods of time is not possible on the rheometer, as this leads to massive dehydration, even with the use of a solvent trap. This made the acquisition of useful data impossible. Therefore, Electrochemical Impedance Spectroscopy (EIS) was used to follow the polymerisation over the course of 14 d. The use of EIS is possible, as the gel formation depletes the solution of positively charged monomers and immobilises them in the polymer network. Consequently, the impedance *Z* increases as the polymerisation progresses. From that, the real part *Z*_real_ can be calculated (Figure 6).

Immediately after initiation, all four solutions show a similar resistance of *Z*_real_ ≈ 6 Ω, as this depends only on the starting concentration. The slight differences are due to the different sizes of the cross-linkers, and the solutions containing TAAB show the lowest *Z*_real_, as this is presumed to have the highest mobility. Initially, Z_real_ increases for all four samples in accordance with the theory. Following that, the reaction mixture containing BIS continues to show the expected behaviour in the form of a saturation curve, which levels off after approx. 7 d. This indicates continued monomer depletion of the solution. However, the curves of pure DADMAC and the samples containing the allyl-based cross-linkers 1a–c exhibit a sharp bend after significantly less than a day. In terms of the measured quantity *Z*_real_ this means that the number of ions stays rather constant. Transferred to the polymerisation this means that the conversion of monomer has subsided or proceeds only at a very low rate. This seems to contradict the rheological measurements in as far as e. g. for TAAB (1a), the build-up of G’ is still accelerating after approx. 1 d (cf. Figure 5 and description above). Apart from BIS and the other cross-linkers 1a–c, one reason for the difference between rheological measurements and EIS could be that the mechanism postulated in Figure 4 holds true, to a certain extent, for all allyl-based cross-linkers. In that case, the DADMAC monomer and the cross-linkers are first incorporated into largely linear chains with only little cross-linking. This is possible if the rate of polymerisation of DADMAC and the first two allyl units of the cross-linkers 1a–c is similar as postulated above. The ratio between chain growth and cross-linking appears to vary between 1a, b, and c as the build-up of *G*’ occurs at different rates (Figure 5) and the *Z*_real_ curves do not run on top of each other. After larger amounts of the low-molecular weight monomers have been immobilised, polymerisation shifts increasingly to the cross-linking reaction. This can only be detected with difficulty by the EIS, as the number of mobile charges does not change much. Cationic hydrogels are still highly electrically conductive, even in deionised water [24]. The increasing viscosity, as recorded by the rheometer, additionally hampers the movement of remaining charges and together this accounts for the slight and steady increase of *Z*_real_ in Figure 6. However, the final gels appear to contain approx. 7–9 % residual monomer. In contrast, samples containing BIS incorporate much more cross-linker—and less DADMAC—in early stages of the reaction, which leads to much higher viscosities after shorter times. This limits the mobility of the leftover ions, which accounts for the increase in *Z*_real_ and decreasing rates of polymerisation. As a consequence, DADMAC consumption continues over longer periods of time.

**Figure 7:**
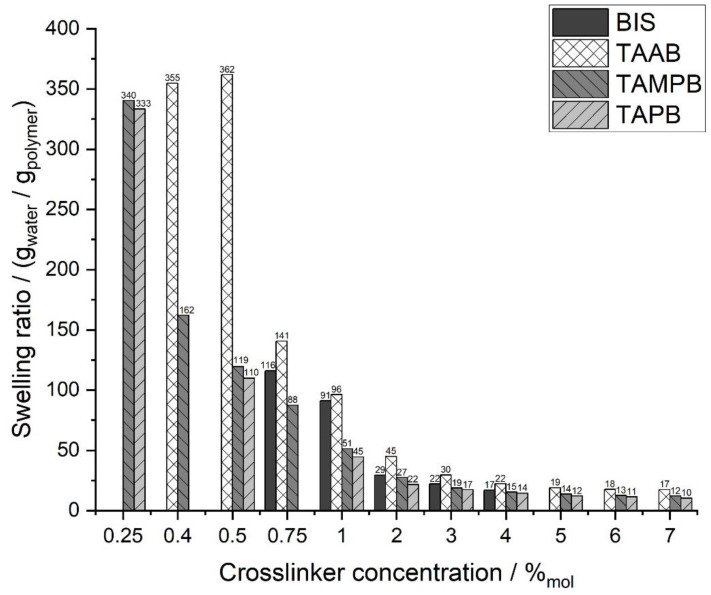
Swelling ratios of cationic poly(DADMAC) gels with different cross-linkers as a function of the cross-linker concentration. Reproducibility is approx. ±5 % for values <200 g/g and approx. ±14 % above that.

Based on these findings, gels used for the swelling tests were allowed to react for 14 days in order to ensure extensive polymerisation. The cross-linkers were used in ratios of 0.25 mol% to 7 mol% (Figure 7). However, gels with low ratios of TAAB (<0.4 %) or BIS (<0.75 %) were not considered. Although these appear to be coherent during synthesis, they dissolve in double-distilled water. For TAAB, this is another indication of the low crosslinking efficiency, which was already assumed above. As the copolymerisation of DADMAC with BIS is considered to form inhomogeneous networks, very low amounts of BIS in the sample will be quickly consumed at the beginning of the reaction so that the “network” formed later contains little to no cross-links. The highly cross-linked parts appear to be smaller than the mesh size of tea-bags (90 μm), and are lost during the swelling experiments. As mentioned before, gels with > 4 mol% BIS could not be prepared due to the solubility limit.

In general, the swelling capacity decreased with increasing the cross-linker content up to approx. 5 mol%, after which it remained constant at approx. 10 to 18 g/g depending on the actual cross-linker. Gels based on the allyl cross-linkers, particularly 1b, c, could be synthesized with very low amounts of cross-linker, giving rise to very high swelling capacities of up to 360 g/g. Such a significant increase in the degree of swelling at low crosslinking densities has already been observed for other crosslinked hydrogel systems [25]. These values are comparable to common acrylate superabsorbent polymers [26,27], and are another indication of the homogeneous nature of these gels. In contrast, the coherent gel with the lowest concentration of BIS could only absorb 116 g/g.

**Figure 8:**
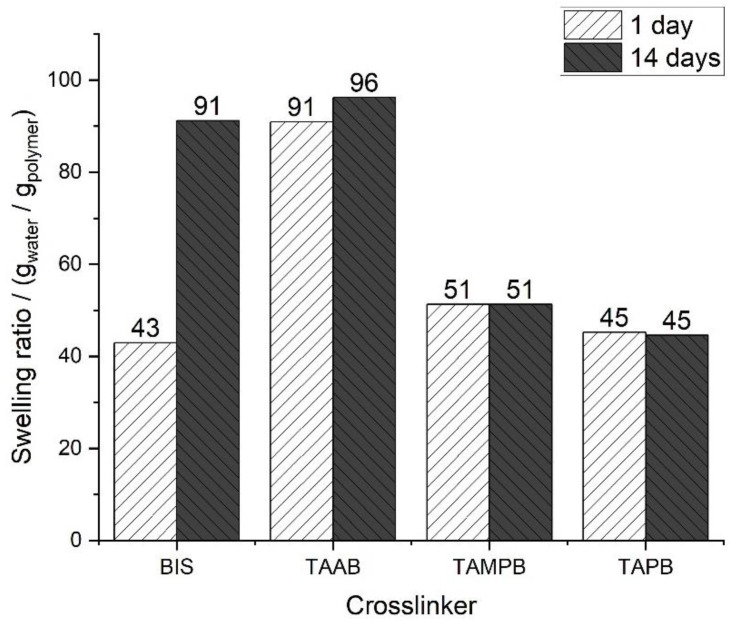
Swelling ratios of cationic poly(DADMAC) gels containing 1 mol% of the cross-linkers 1a-c and BIS after 1 day and 14 days polymerisation time.

Finally, to test whether the assumption that BIS leads to inhomogeneous networks is true, gels containing 1 mol% of the cross-linkers 1a–c and BIS were polymerised for only 1 d and their swelling behaviour was compared to that in Figure 7 (polymerisation time 14 d). Gels containing the allyl-based cross-linkers 1a–c showed virtually no difference in the swelling capacity, whereas for those containing BIS, the value doubled (Figure 8). This strongly supports the postulated inhomogeneous network. As outlined above, the reactivity ratios indicate the preferred incorporation of BIS into the network. At low conversions, this leads to more strongly cross-linked structures which exhibit lower swelling capacities. This also depletes the solution of BIS so that the network formed at later stages will have increasingly lower cross-linking densities, but at the same time higher swelling capacities. In total, the BIS gels are highly inhomogeneous with increasing swelling capacity over time.

## 3. Conclusions

The three tetraallylammonium-based cross-linkers tetraallylammonium bromide, *N*,*N*,*N’*,*N’*-tetraallylpiperazinium dibromide and *N*,*N*,*N’*,*N’*-tetraallyltrimethylene dipiperidine dibromide are excellent alternatives to the commonly used *N*,*N*’-methylenebisacrylamide for the cross-linking polymerisation of slow monomers such as *N*,*N*-diallyldimethylammonium chloride and similar monomers in water. In these cases, the incorporation of monomers into the network proceeds statistically. The combination of rheological and EIS data indicates that, initially, largely linear chains with little cross-linking form, and that cross-linking is a slower process due to the formation of spiro structures. Both factors favour the formation of homogeneous networks. Homogeneity is a prerequisite for the formation of coherent gels with low cross-linking densities, i.e., high swelling capacities, as well as firm, resilient gels. The former have potential applications in biomedical and hygiene products, while the latter are more suitable as ion-exchange resins and in soil applications.

## 4. Materials and Methods

### 4.1. Materials

The anion exchange-resin Lewatit Monoplus MP 800 was provided by Lanxess (Leverkusen, Germany). Double-distilled water, potassium hydroxide, chloroform (≥99%), acetone (≥99%), dichloromethane (≥99%), potassium persulfate, sodium metabisulphite, sodium hydroxide (97%) and *N,N’*-methylenebisacrylamide were obtained from VWR International GmbH (Darmstadt, Germany). Potassium carbonate (99%), allyl bromide (99%), methanol (99%) and 1,3-bis(4-piperidyl)propane (97+%) were obtained from Alfa Aesar (Kandel, Germany), diallyldimethylammonium chloride (65 wt% in H_2_O) and triallylamine (99%) were obtained from Sigma Aldrich. Piperazine, diallylamine (97%) and D_2_O (99.9%) were purchased from Merck KGaA (Darmstadt, Germany). The polyester filter bags with a maximum mesh size of 90 µm were bought from Rosin Tech Products (Bethpage, NY, USA).

### 4.2. Syntheses

#### 4.2.1. Preparation of Tetraallylammonium Bromide 1a (TAAB)

A solution of 7.41 mL (6.00 g, 43.72 mmol) triallylamine in 50 mL acetone was placed in a 250 mL round-bottom flask and cooled in an ice-water bath. To this, 4.53 mL (6.35 g, 52.47 mmol, 1.2 equiv.) allyl bromide were added over the period of one hour. Afterwards, the solution was heated to 88 °C for 48 h. The solution was filtered and the resulting solid was washed several times with acetone. Tetraallyl ammonium bromide was obtained as a white crystalline solid. Both thermogravimetry and differential scanning calorimetry show unanimously that 1a decomposes at 185 °C. Previous reports listed this as melting point [28].

Yield: 6.90 g (61%); Decomposition at 185 °C.

^1^H-NMR (400 MHz, D_2_O) δ 6.09 (m, 4H), 5.81–5.69 (m, 8H), 3.93 (d, *J* = 7.3 Hz, 8H).

(Figure S2 in the Supporting Information).

^13^C-NMR (300 MHz, D_2_O) δ 128.52, 123.98, 60.32.

(Figures S3 and S4 in the Supporting Information).

ATR-IR: 2937 cm^−1^ ν(C-H), 1447 cm^−1^ γ (CH2), 1020 cm^−1^ ν(C-N).

(Figure S5 in the Supporting Information).

#### 4.2.2. Preparation of Diallylpiperazine

10 g (116.1 mmol) of piperazine was dissolved in 31 mL saturated potassium carbonate solution in a 250 mL round-bottom flask. The solution was cooled in an ice-water bath and 22.0 mL (30.8 g, 255 mmol, 2.2 equiv. with regard to piperazine) allyl bromide was slowly added over the course of 1 h. When approx. half of the allyl bromide had been added, the solution separated into a lower white suspension and an upper oily yellow phase formed. After complete addition, the reaction mixture was stirred at room temperature for 72 h before adding 50 mL Dichloromethane. The phases were separated and the aqueous phase was extracted three times with Dichloromethane. The combined organic phases were dried with magnesium sulphate, filtered, and the solvent was removed under reduced pressure. 11.43 g (54%) of the crude diallylpiperazine was obtained as pale, yellow, oily liquid.

^1^H-NMR (400 MHz, Chloroform-d) δ 5.85 (m, 2H), 5.13 (m, 4H), 2.98 (d, *J* = 6.6 Hz, 4H), 2.47 (s, 8H).

(Figure S6 in the Supporting Information).

#### 4.2.3. Preparation of *N,N,N’,N‘*-Tetraallyl Piperazinium Dibromide 1b (TAPB)

11.43 g (68.74 mmol,) diallylpiperazine was dissolved in 95 mL of acetone. To this, 17.82 mL (24.95 g, 206.23 mmol, 3 equivalents) allyl bromide was slowly added. The reaction mixture was refluxed for 72 h at 88 °C. During this, the crude product precipitated as a beige solid, which was filtered, washed with acetone, and recrystallized from methanol to yield 28.06 g (65%) tetraallylpiperazinedibromide as white crystalline solid. Similar to 1a, the dibromide 1b was found to decompose at 210 °C, previously reported as m.p. 207 °C [28])

^1^H-NMR (400 MHz, D_2_O) δ 6.10 (m, 4H), 5.90 (m, 8H), 4.26 (d, *J* = 7.2 Hz, 8H), 3.99 (s, 8H).

(Figure S7 in the Supporting Information).

^13^C-NMR (300 MHz, D_2_O) δ 130.78, 122.22, 50.66.

(Figures S8 and S9 in the Supporting Information).

ATR-IR: 2988 cm^−1^ ν(C-H), 1473 cm^−1^ γ (CH2), 1150 cm^−1^ ν(C-N).

(Figure S10 in the Supporting Information).

#### 4.2.4. Preparation of *N,N,N‘,N‘*-Tetraallyl Trimethylene Dipiperidine Dibromide 1c (TAMPB)

The preparation follows the two-step procedure outlined for TAPB. 16 g (81.49 mmol) trimethylenedipiperidine afforded 8.51 g (38%) crude diallyltrimethylendipiperidine (DAMP) as yellow, oily liquid.

^1^H-NMR (600 MHz, Chloroform-d) δ 5.82 (m, 2H), 5.07 (m, 4H), 2.91 (d, *J* = 6.7 Hz, 4H), 2.85 (d, *J* = 11.5 Hz, 4H), 1.81 (t, *J* = 11.1 Hz, 4H), 1.60 (d, *J* = 9.5 Hz, 4H), 1.19 (m, 12H).

(Figure S11 in the Supporting Information).

In the second step, 8.51g DAMP yielded after recrystallization in methanol 11.53 g (72%) TAMPB as beige crystalline solid. Similar to 1a, the dibromide 1c was found to decompose at 220 °C.

^1^H-NMR (400 MHz, D_2_O) δ 6.07 (m, 4H), 5.73 (m, 8H), 4.00 (d, *J* = 7.3 Hz, 4H), 3.89 (d, *J* = 7.4 Hz, 4H), 3.52 (d, *J* = 12.2 Hz, 4H), 3.28 (t, *J* = 11.2 Hz, 4H), 1.93 (d, *J* = 9.5 Hz, 4H), 1.68 (m, 6H), 1.40 (m, 6H).

(Figure S12 in the Supporting Information).

^13^C-NMR (300 MHz, D_2_O) δ 128.61, 128.22, 123.77, 65.22, 56.14, 34.31, 32.15, 25.25, 22.51.

(Figures S13 and S14 in the Supporting Information).

ATR-IR: 3013 cm^−1^ ν(C-H), 1497 cm^−1^ γ (CH2), 1037 cm^−1^ ν(C-N).

(Figure S15 in the Supporting Information).

#### 4.2.5. Copolymerisation of Crosslinked DADMAC-Hydrogels

The method is described using 2 mol% crosslinker as an example. Details on the compositions and further experimental details can be found in Table S1 in the Supporting Information. A mixture of 5 g of a DADMAC solution (65 wt% in water, 20 mmol), 30 mg sodium disulfite (0.16 mmol), and 62 mg *N*,*N*’-methylenebisacrylamide (0.4 mmol, 2 mol% related to the DADMAC content) was stirred until the crosslinker had completely dissolved. Meanwhile, 60 mg KPS were dissolved separately in 1.5 mL H_2_O and then added to the monomer solution. The mixtures containing **1a–c** were stirred for 10 min and those with BIS for shorter times, as gelation with BIS begins earlier. An example image of a swollen polyDADMAC gel is given in the Supporting Information (Figure S21).

### 4.3. Infrared Spectroscopy (FTIR-ATR)

Infrared spectra were recorded using a Perkin Elmer Spectrum Two UATR FTIR spectrometer equipped with a diamond ATR (attenuated total reflection) window. All spectra were recorded in the spectral range of 4000–400 cm^−1^ with 12 scans at a spectral resolution of 4 cm^−1^. Before each measurement, the diamond ATR crystal was cleaned with isopropanol.

### 4.4. Rheology

The rheological data were recorded on an Anton Paar Modular Compact Rheometer 102. A plate-plate geometry with a diameter of 25 mm made of stainless steel was used. All samples were measured with a plate gap of 1 mm at 20 °C. The isothermal oscillating time-dependent measurements were performed at 1% amplitude and an angular frequency of 1 Hz over a period of 16 h. The set-up was covered with a solvent trap over this period.

Sample preparation follows the above procedure for the preparation of DADMAC hydrogels. After adding the KPS initiator, the mixture was stirred intensively for 5–10 min (the exact time was noted and used in the calculation of the gel point), depending on the rate of polymerisation. 560 μL of this mixture was then transferred to the rheometer using an Eppendorf pipette. The gel point was calculated from the intersection of the *G*’ and *G*’’ curves (determined graphically from recorded data) [29,30], by adding the stirring and transfer time. Details for the determination of the gel points can be found in Table S2 in the Supporting Information. An example of the graphical determination of the gel point is given in the Supporting Information (Figure S1). The measurements for the samples with 2 mol% of the different crosslinkers were repeated twice. The determined gel points deviated by approx. ±10%.

### 4.5. Electrochemical Impedance Spectroscopy (EIS)

Impedance spectra were recorded on a Gamry Potentiostat 400 (C3 Prozess und Analysetechnik GmbH, Munich, Germany). Sample preparation followed the above procedure for the preparation of crosslinked DADMAC hydrogels using 1 mol% of the desired crosslinker BIS, TAAB, TAPB, or TAMPB). After adding the KPS initiator and stirring intensively for 10 min, the mixtures were then transferred to standard electroporation cuvettes (Gap width = 4 mm), equipped with two opposing aluminium electrodes of 9 × 18 mm^2^ (blocking electrodes). The solution was covered with a layer of silicon oil to exclude oxygen and prevent evaporation. Over a course of 16 days, the impedance *Z* was recorded every hour using a voltage of 141 mV. As shown in Figure S20, the phase angle φ = 90° at an ac frequency of 100 kHz and with

$$Z=Z_{real}+iZ_{imag}=\left|Z\right|e^{i\varphi }$$

follows that *Z*_imag_ ≈ 0 and *Z* = *Z*_real_.

### 4.6. Swelling Experiments (Teabag Tests)

Approx. 100 mg of lyophilized hydrogel were weighed into polyester filter-bags and submerged in 0.5 L of double-distilled water at 22 °C. The mass increase was initially recorded every 15 min, later every 30 min to one hour, depending on the previous mass changes. After removing the teabags from the solutions, they were carefully stripped and hung up for 5 min to drain the nonabsorbed liquid. The reported maximum swelling ratios were determined by averaging the last three recorded values. The swelling experiments were each repeated once. It was found that the determined values varied by ±5% at low degrees of swelling. For high degrees of swelling (>200 g/g), deviations of up to ±14 % were found.

### 4.7. NMR-Spectroscopy

^1^H-NMR and ^13^C-NMR spectra were recorded on a Mercury 400 spectrometer (Varian, Palo Alto, CA, USA). Chemical shifts were calculated using the HDO signal at 4.64 ppm or the CDCL_3_ signal at 7.26 ppm as a reference.

## Data Availability

Data is available from the authors upon request.

## Supplementary Materials

The following are available online at https://www.mdpi.com/article/10.3390/gels8020100/s1, Figure S1: Example of the rheological determination of G’ and G’’ of a DADMAC gelation mixture, Figure S2: ^1^H-NMR-spectrum (400 MHz, D_2_O) of Tetraallyl ammonium bromide (TAAB), Figure S3: ^13^C-NMR-spectrum (400 MHz, D_2_O) of Tetraallyl ammonium bromide (TAAB), Figure S4: ^13^C-NMR-APT-spectrum (400 MHz, D_2_O) of Tetraallyl ammonium bromide (TAAB), Figure S5: ATR-IR-spectrum of Tetraallyl ammonium bromide (TAAB), Figure S6: ^1^H-NMR-spectrum (400 MHz, CDCl_3_) of *N*,*N*‘-diallyl piperazine (DAP), Figure S7: ^1^H-NMR-spectrum (400 MHz, D_2_O) of *N,N,N’,N‘*-tetraallyl piperazinium dibromide (TAPB), Figure S8: ^13^C-NMR-spectrum (400 MHz, D_2_O) of *N,N,N’,N‘*-tetraallyl piperazinium dibromide (TAPB), Figure S9: ^13^C-NMR-APT-spectrum (400 MHz, D_2_O) of *N,N,N’,N‘*-tetraallyl piperazinium dibromide (TAPB), Figure S10: ATR-IR-spectrum of *N,N,N’,N‘*-tetraallyl piperazinium dibromide (TAPB), Figure S11: ^1^H-NMR-spectrum (400 MHz, CDCl_3_) of *N*,*N*‘-diallyl trimethylene dipiperidine (DAMP), Figure S12: ^1^H-NMR-spectrum (400 MHz, D_2_O) of *N*,*N*,*N*‘,*N*‘-tetraallyl trimethylene dipiperidine dibromide(TAMPB), Figure S13: ^13^C-NMR-spectrum (400 MHz, D_2_O) of *N*,*N*,*N*‘,*N*‘-tetraallyl trimethylene dipiperidine dibromide(TAMPB), Figure S14: ^13^C-NMR-APT-spectrum (400 MHz, D_2_O) of *N*,*N*,*N*‘,*N*‘-tetraallyl trimethylene dipiperidine dibromide(TAMPB), Figure S15: ATR-IR-spectrum of *N*,*N*,*N*‘,*N*‘-tetraallyl trimethylene dipiperidine dibromide(TAMPB), Figure S16: Copolymerisation diagram of poly(DADMAC-co- *N*,*N*’-Methylenebisacrylamide) calculated according to alfrey and price, Figure S17: ^1^H-NMR-spectra (400 MHz, D_2_O) pre-swollen in D_2_O, Figure S18: ^13^C-NMR-spectra(400 MHz, D_2_O) pre-swollen in D_2_O, Figure S19: ^1^H-NMR-spectrum (400 MHz, D_2_O) of Poly-tetraallyl ammonium bromide (pTAAB), Figure S20: Exemplary bodeplot of a DADMAC gelation mixture with 1 mol % TAMPB after 10 h of polymerisation, Figure S21: Poly(DADMAC) crosslinked with 1%w.t. BIS and swollen in double distilled Water. Table S1: Details on the sample composition used to prepare crosslinked DADMAC hydrogels, Table S2: Details on the determination of the gelation points of crosslinked DADMAC hydrogels.
