# Supplementary material for: Multivalent Allylammonium-Based Cross-Linkers for the Synthesis of Homogeneous, Highly Swelling Diallyldimethylammonium Chloride Hydrogels
Source: Gels. 2022 Feb 8;8(2):100. doi: 10.3390/gels8020100 (PMC8871358; doi:10.3390/gels8020100)
Supplement: Supplementary file 1 [file gels-08-00100-s001.zip › gels-1510912-supplementary.pdf]

Supplementary Materials:

To

Multivalent ammonium bromide based crosslinkers for the synthesis of diallyldimethylammonium chloride hydrogels

Tim Mrohs [1] and Oliver Weichold[2,*]

[1] Institute of Building Materials Research, RWTH Aachen University, Schinkelstraße 3, 52062 Aachen, Germany.
* Correspondence: weichold@ibac.rwth-aachen.de

Table S-1: Details on the sample composition used to prepare crosslinked DADMAC hydrogels**.**

	Crosslinker				
**Sample**	**Type**	**ratio / mol-%**	**n / mmol**	**m / mg**	**Stirring time / min**
BIS0,25	BIS	0,25	0,05	7,8	10
BIS0,5	BIS	0,5	0,1	15,5	10
BIS1	BIS	1	0,2	31	5
BIS2	BIS	2	0,4	62	5
BIS3	BIS	3	0,6	93	1
BIS4	BIS	4	0,8	124	1
BIS5	BIS	5	1	155	1
TAAB0,25	TAB	0,25	0,05	13	10
TAAB0,5	TAB	0,5	0,1	26	10
TAAB1	TAB	1	0,2	51,9	10
TAAB2	TAB	2	0,4	103,2	10
TAAB3	TAB	3	0,6	155,7	10
TAAB4	TAB	4	0,8	207,6	10
TAAB5	TAB	5	1	259,5	10
TAAB6	TAB	6	1,2	311,4	10
TAAB7	TAB	7	1,4	363,3	10
TAPB0,25	TAP	0,25	0,05	20,5	10
TAPB0,5	TAP	0,5	0,1	41,1	10
TAPB1	TAP	1	0,2	81,2	10
TAPB2	TAP	2	0,4	162,4	10
TAPB3	TAP	3	0,6	243,6	5
TAPB4	TAP	4	0,8	324,8	5
TAPB5	TAP	5	1	406,0	5
TAPB6	TAP	6	1,2	487,2	5
TAPB7	TAP	7	1,4	568,4	5
TAMPB0,25	TAMP	0,25	0,05	26,8	10
TAMPB0,5	TAMP	0,5	0,1	53,7	10
TAMPB1	TAMP	1	0,2	107,3	10
TAMPB2	TAMP	2	0,4	214,6	10
TAMPB3	TAMP	3	0,6	321,9	10
TAMPB4	TAMP	4	0,8	429,2	10
TAMPB5	TAMP	5	1	536,5	10
TAMPB6	TAMP	6	1,2	643,8	10
TAMPB7	TAMP	7	1,4	751,1	10

Table S-2: Details on the determination of the gelation points of crosslinked DADMAC hydrogels.

Sample	Crosslinker		Stirring time / min	Preparation time / min	Recorded gel Point[a] / min
	Type	ratio / mol-%			
1	BIS	1	5	1	7,7
2	BIS	2	5	1	1,6
3	BIS	3	1	1	2,7
4	BIS	4	1	1	1,4
5	TAPB	1	10	1	85
6	TAPB	2	10	1	11
7	TAPB	3	5	1	12
8	TAPB	5	5	1	8,5
9	TAMPB	1	10	1	124
10	TAMPB	2	10	1	110
11	TAMPB	3	10	1	87
12	TAMPB	5	10	1	48
13	TAAB	1	10	1	1740
14	TAAB	2	10	1	1170
15	TAAB	3	10	1	340
16	TAAB	5	10	1	69

[a] The recorded gel points were taken graphically from the plotted values of the storage (G′) and loss (G′′) modulus against time. Data points were taken every 10 seconds. The time at which the values of G' > G'' remained was determined as the value for the gel point by adding the stirring and transfer time, exemplarily shown in figure.S1.

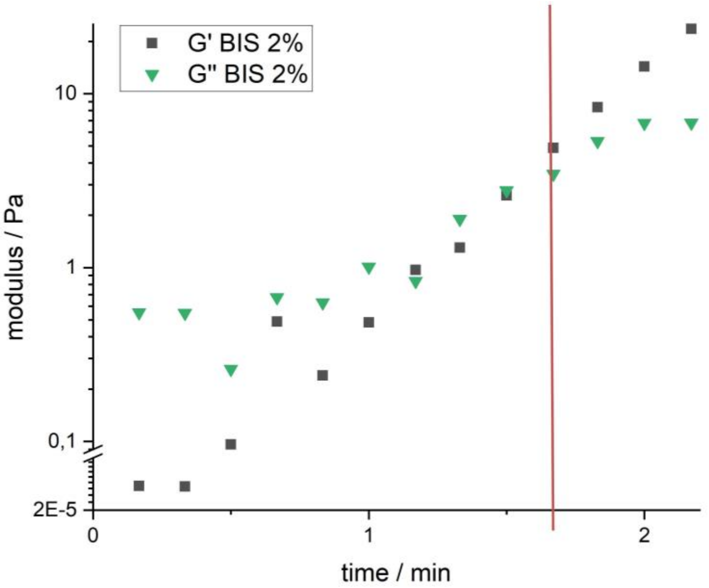

Figure S1. Example of the rheological determination of G' and G'' of a DADMAC gelation mixture with 2 mol-% BIS for the graphical determination of the gel point. The real gel point (7.6 min) results from the read gel point (1.6 min) + stirring time (5 min) + preparation time (1 min).

Tetraallyl ammonium bromide (TAAB)

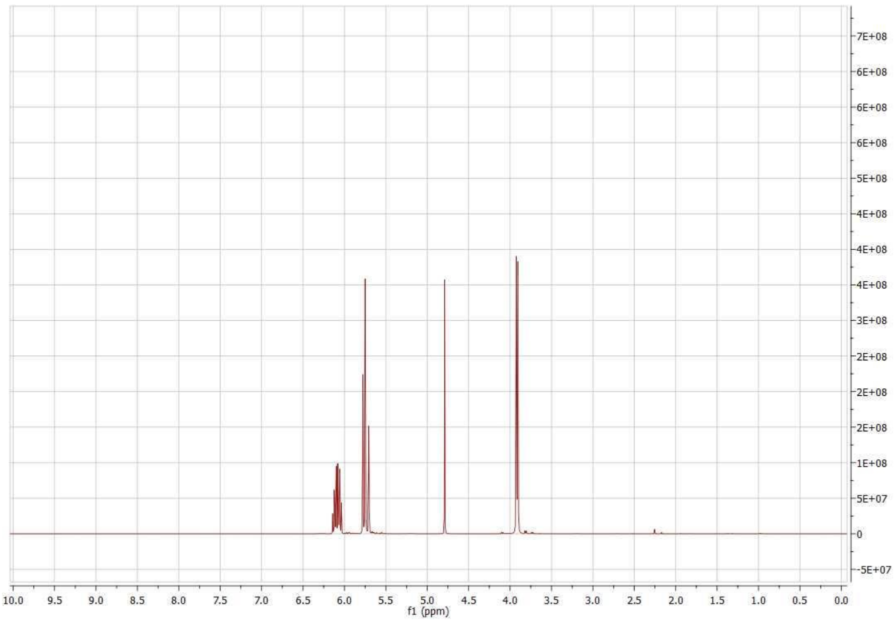

Figure S2. ^{1}H-NMR-spectrum (400 MHz, D_2O) of Tetraallyl ammonium bromide (TAAB).

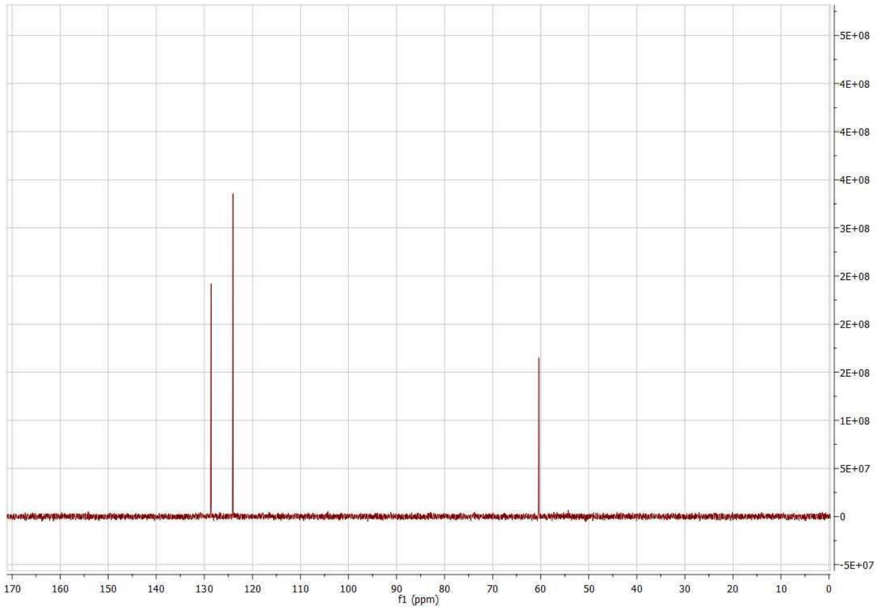

Figure S3. ^{13}C-NMR-spectrum (400 MHz, D_2O) of Tetraallyl ammonium bromide (TAAB).

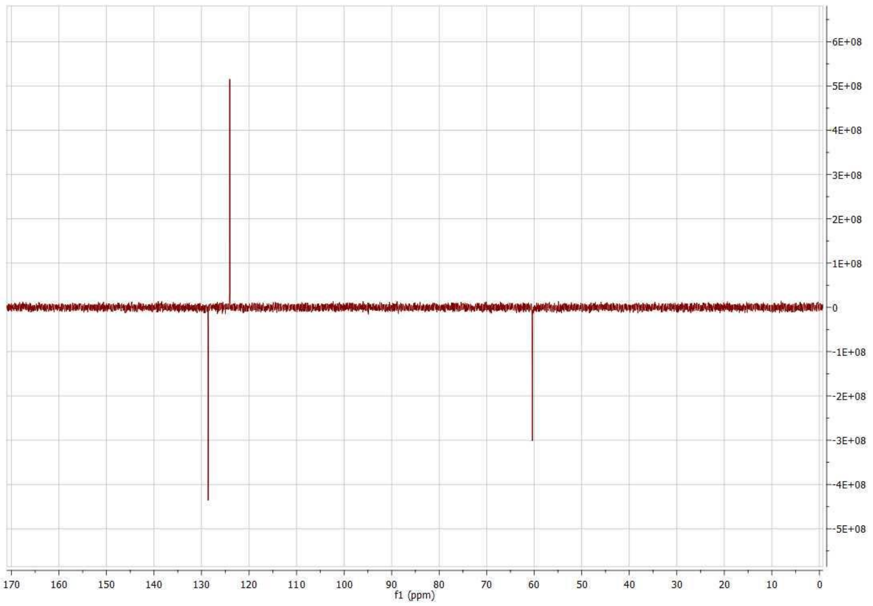

Figure S4. ^{13}C-NMR-APT-spectrum (400 MHz, D_2O) of Tetraallyl ammonium bromide (TAAB).

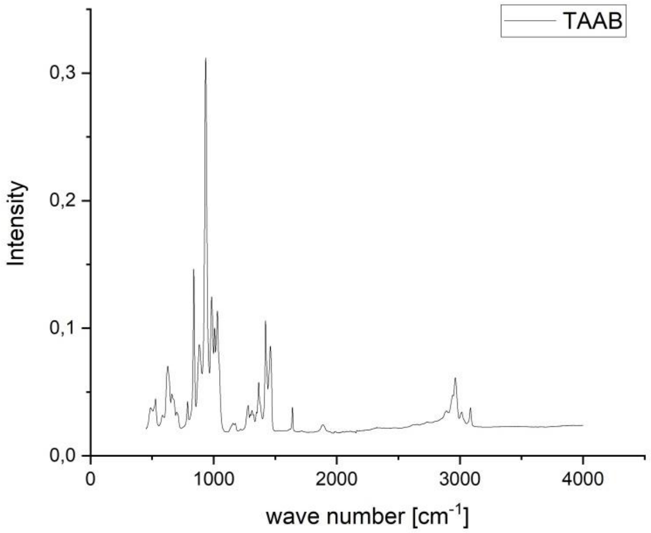

Figure S5. ATR-IR-spectrum of Tetraallyl ammonium bromide (TAAB).

N,*N'*-diallyl piperazine (DAP)

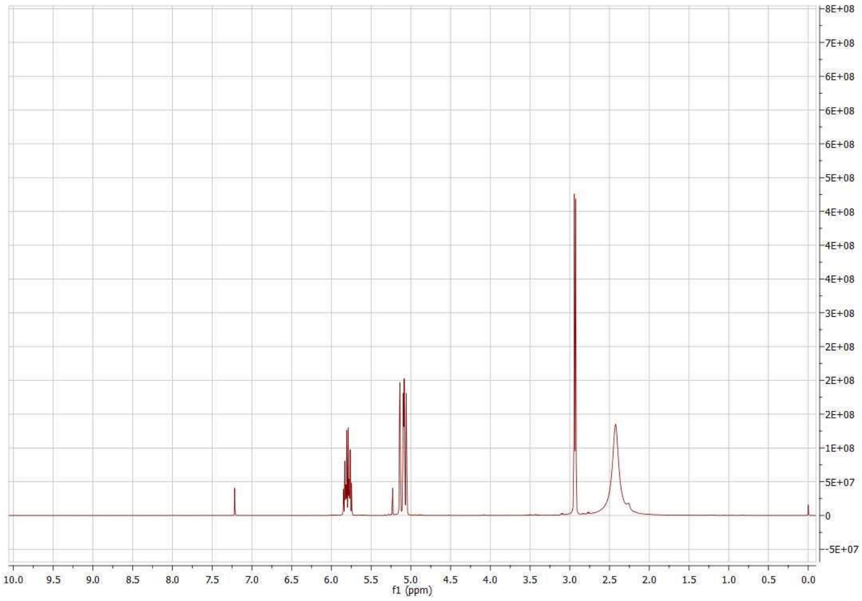

Figure S6. ^{1}H-NMR-spectrum (400 MHz, $CDCl_3$) of *N*,*N'*-diallyl piperazine (DAP).

N,*N*,*N'*,*N'*-tetraallyl piperazinium dibromide (TAPB)

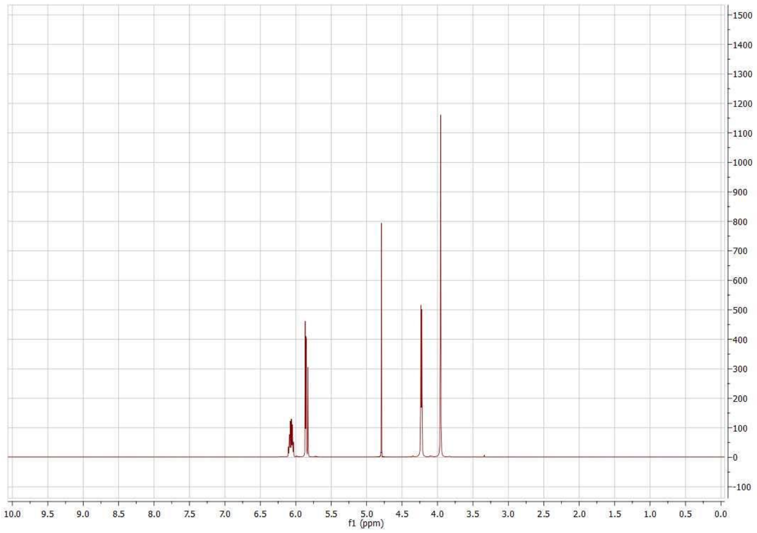

Figure S7. ^{1}H-NMR-spectrum (400 MHz, D_2O) of *N*,*N*,*N'*,*N'*-tetraallyl piperazinium dibromide (TAPB).

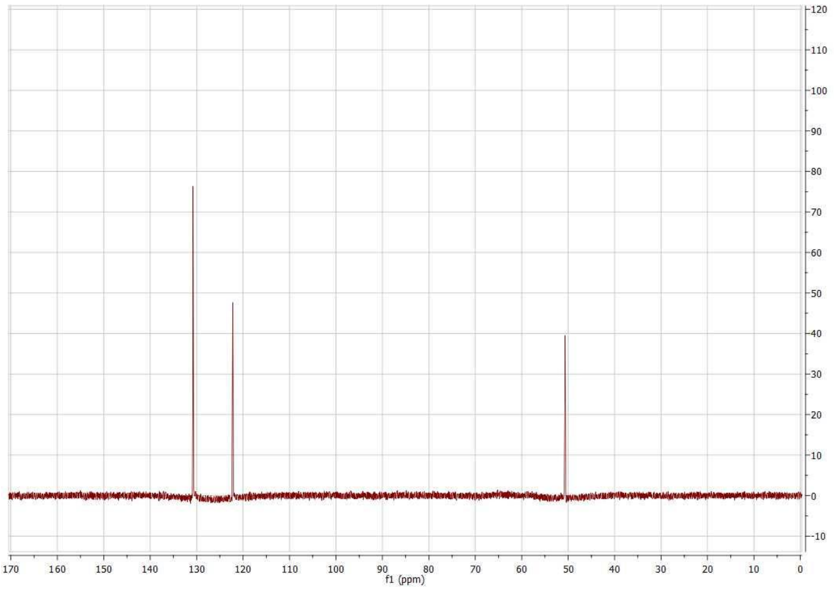

Figure S8. ^{13}C-NMR-spectrum (400 MHz, D_2O) of *N,N,N′,N′*-tetraallyl piperazinium dibromide (TAPB).

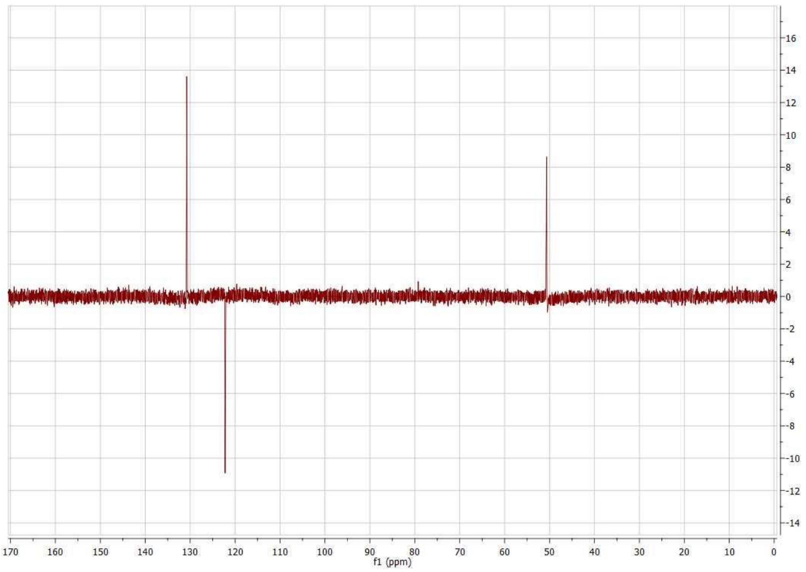

Figure S9. ^{13}C-NMR-APT-spectrum (400 MHz, D_2O) of *N,N,N′,N′*-tetraallyl piperazinium dibromide (TAPB).

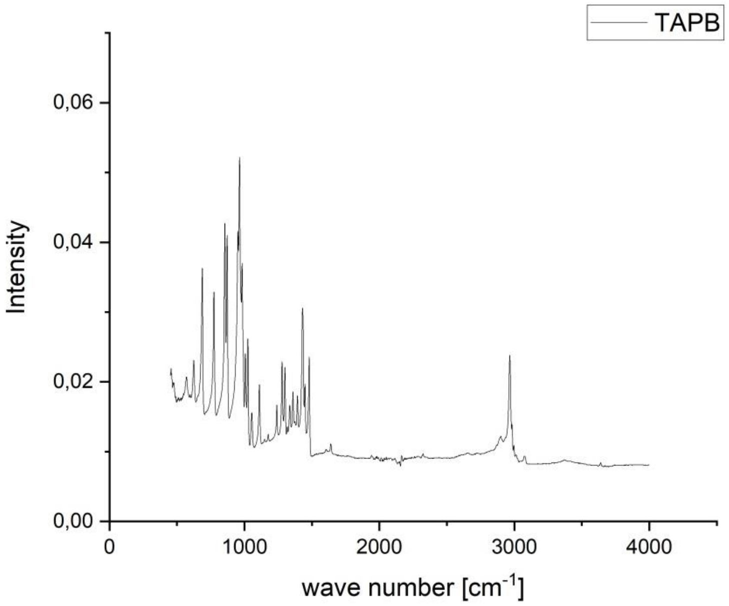

Figure S10. ATR-IR-spectrum of *N,N,N′,N′*-tetraallyl piperazinium dibromide (TAPB).

N,N'-diallyl trimethylene dipiperidine (DAMP)

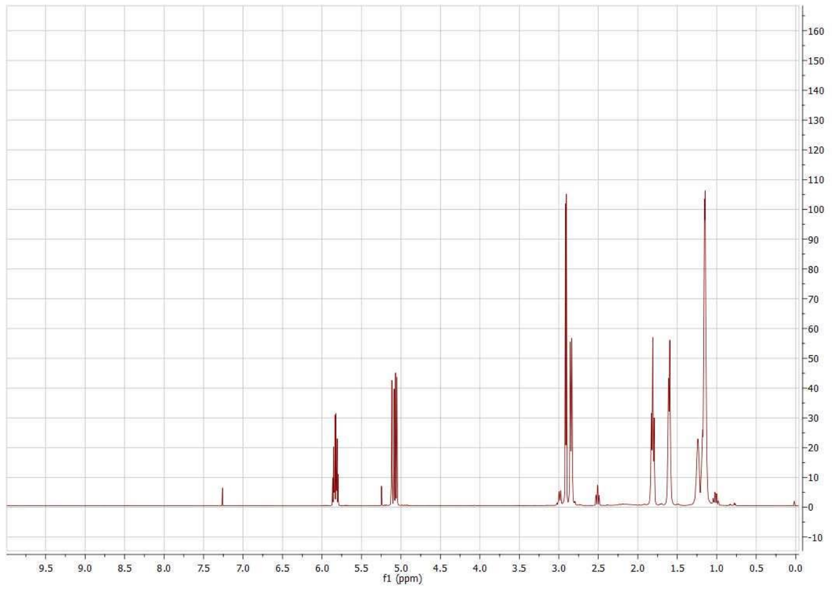

Figure S11. ^{1}H-NMR-spectrum (400 MHz, $CDCl_3$) of *N,N'*-diallyl trimethylene dipiperidine (DAMP).

N,*N*,*N*‘,*N*‘-tetraallyl trimethylene dipiperidine dibromide(TAMPB)

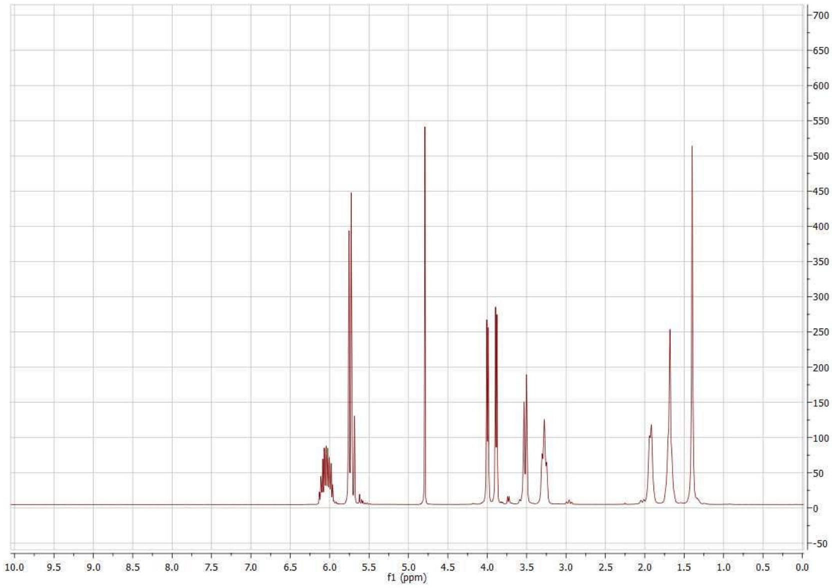

Figure S12. ^{1}H-NMR-spectrum (400 MHz, D_2O) of *N*,*N*,*N*‘,*N*‘-tetraallyl trimethylene dipiperidine dibromide(TAMPB).

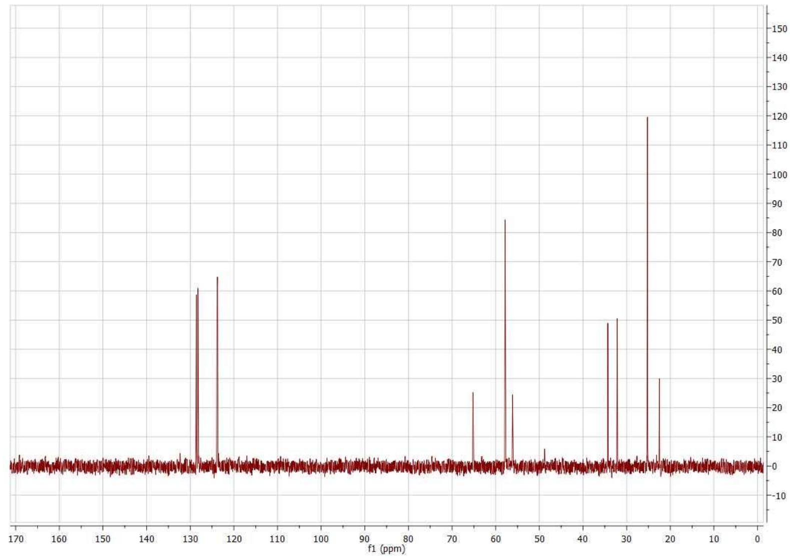

Figure S13. ^{13}C-NMR-spectrum (400 MHz, D_2O) of *N*,*N*,*N*‘,*N*‘-tetraallyl trimethylene dipiperidine dibromide(TAMPB).

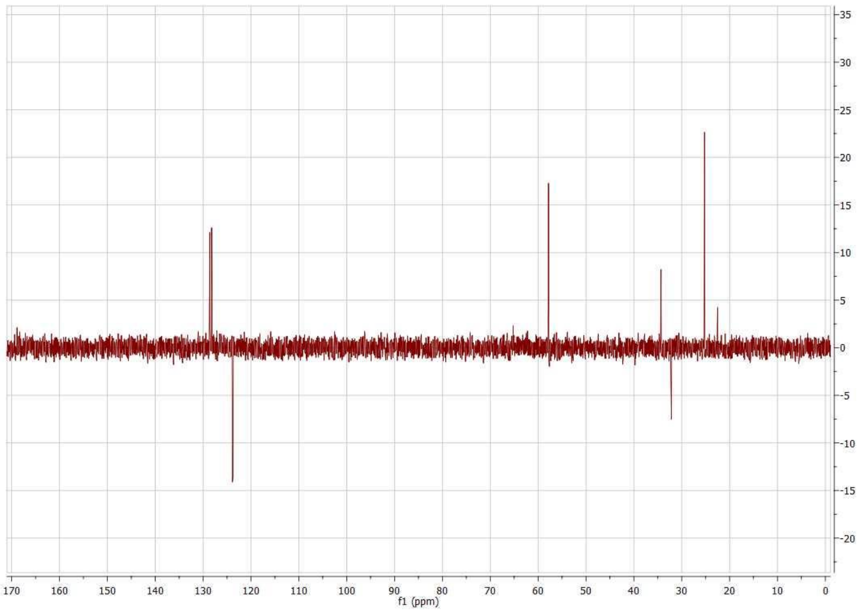

Figure S14. ^{13}C-NMR-APT-spectrum (400 MHz, D_2O) of of *N*,*N*,*N*′,*N*′-tetraallyl trimethylene dipiperidine dibromide(TAMPB).

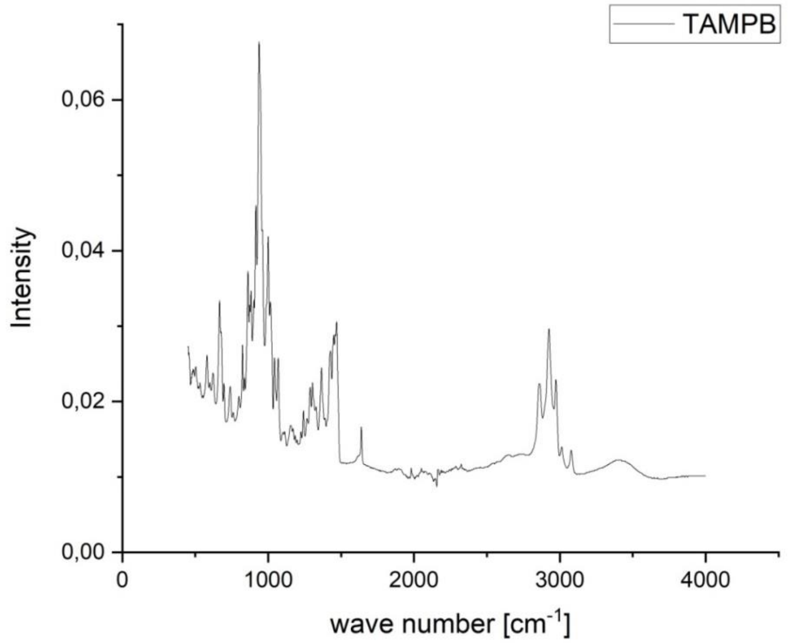

Figure S15. ATR-IR-spectrum of *N*,*N*,*N*′,*N*′-tetraallyl trimethylene dipiperidine dibromide(TAMPB).

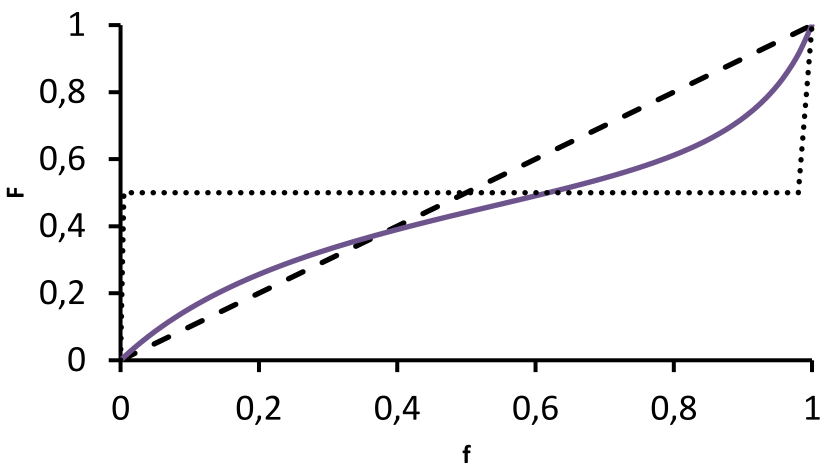

Figure S16. Copolymerisation diagram of poly(DADMAC-co- *N*,*N*'-Methylenebisacrylamide) calculated according to ALFREY and PRICE.

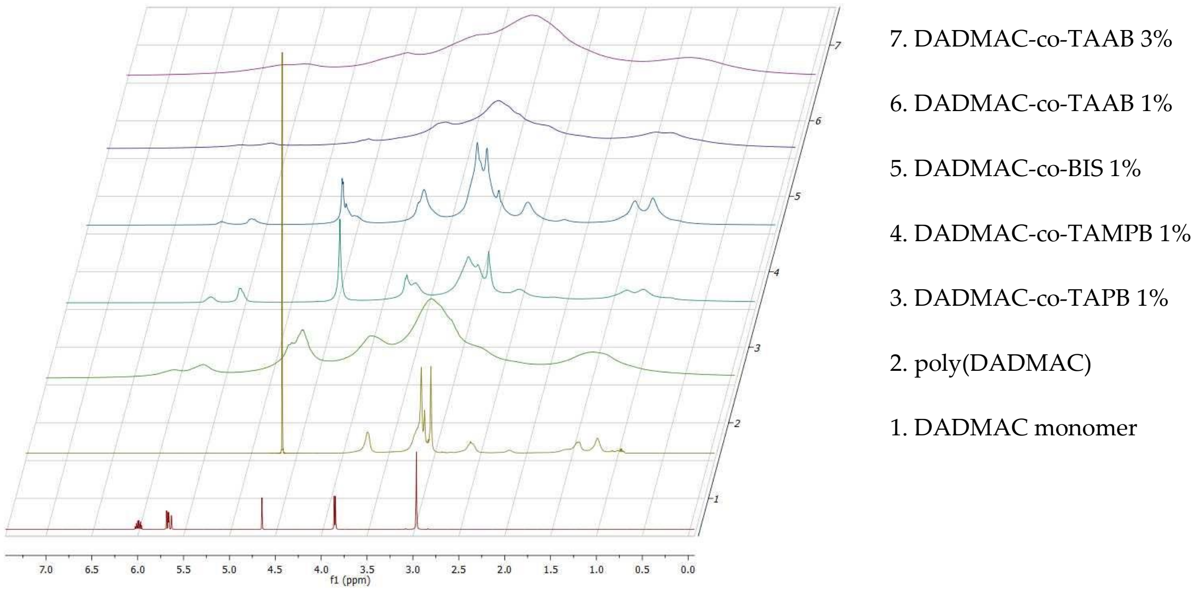

Figure S17. ^{1}H-NMR-spectra (400 MHz, D_2O) of 1) DADMAC Monomer, 2) poly(DADMAC), 3) DADMAC-co-TAPB (1 mol-% crosslinker), 4) DADMAC-co-TAMPB (1 mol-% crosslinker), 5) DADMAC-co-BIS (1 mol-% crosslinker), 6) DADMAC-co-TAAB (1 mol-% crosslinker), 7) DADMAC-co-TAAB (3 mol-% crosslinker) pre-swollen in D_2O.

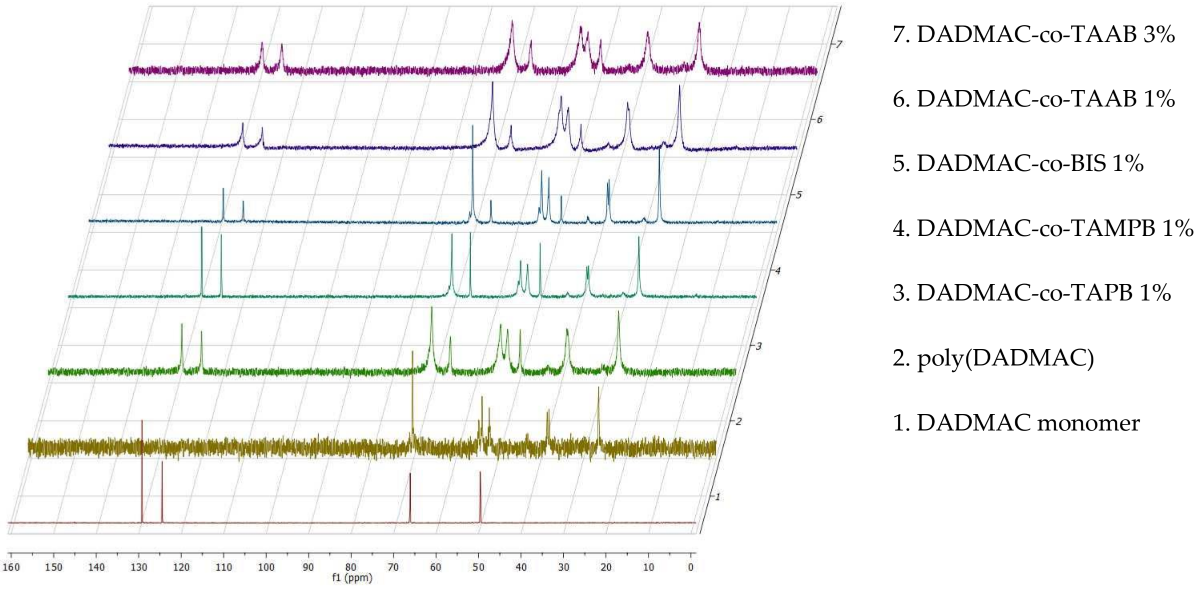

Figure S18. ^{13}C-NMR-spectra(400 MHz, D_2O) of 1) DADMAC Monomer, 2) poly(DADMAC), 3) DADMAC-co-TAPB (1 mol-% crosslinker), 4) DADMAC-co-TAMPB (1 mol-% crosslinker), 5) DADMAC-co-BIS (1 mol-% crosslinker), 6) DADMAC-co-TAAB (1 mol-% crosslinker), 7) DADMAC-co-TAAB (3 mol-% crosslinker) pre-swollen in D_2O.

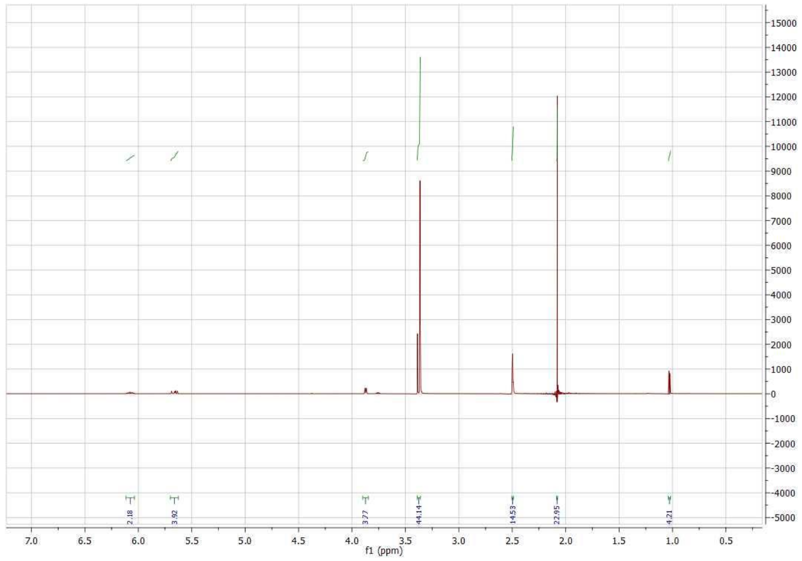

Figure S19. ^{1}H-NMR-spectrum (400 MHz, D_2O) of Poly-tetraallyl ammonium bromide (pTAAB).

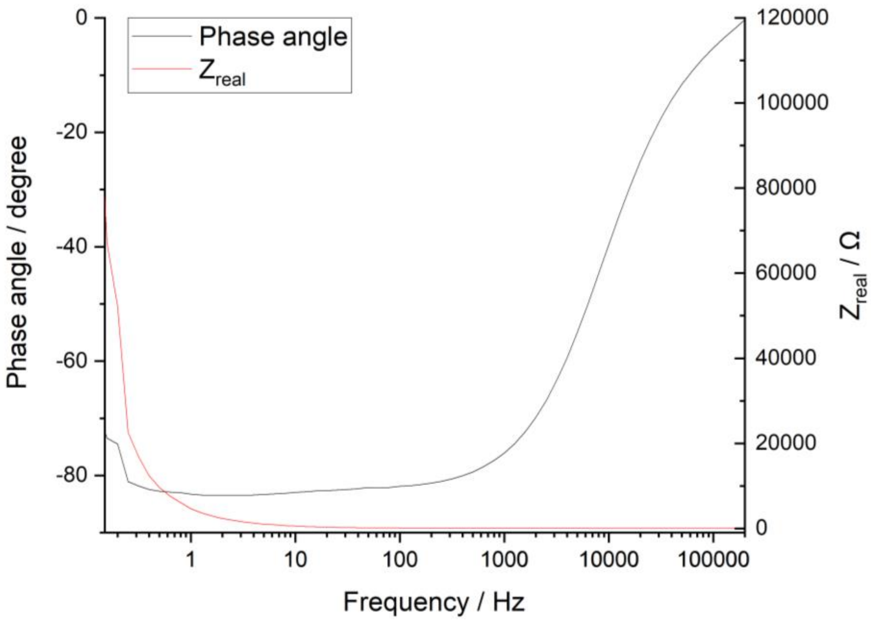

Figure S20. Exemplary bodeplot of a DADMAC gelation mixture with 1 mol-% TAMPB after 10 hours of polymerisation.

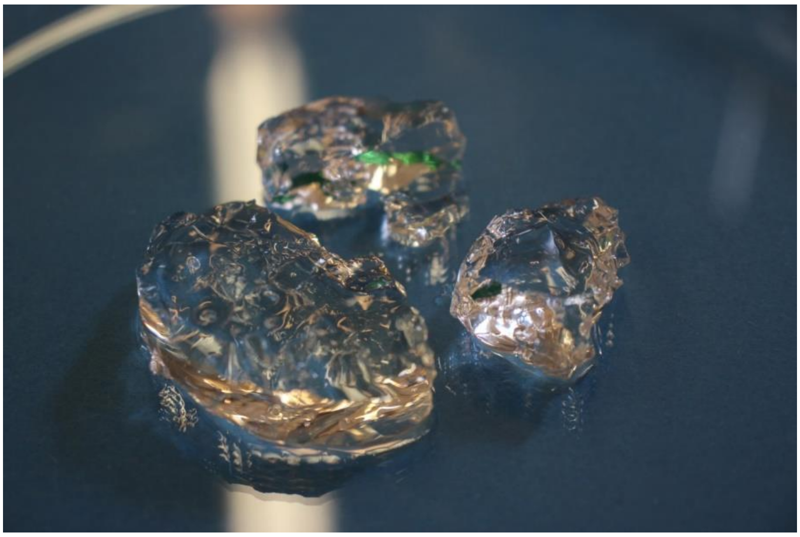

Figure S21. Poly(DADMAOH) crosslinked with 1%w.t. BIS and swollen in double distilled Water.
